# Disability and post-traumatic stress symptoms in the Ukrainian General Population during the 2022 Russian Invasion

**DOI:** 10.1017/S204579602300015X

**Published:** 2023-04-18

**Authors:** Tarandeep S. Kang, Robin Goodwin, Yaira Hamama-Raz, Elazar Leshem, Menachem Ben-Ezra

**Affiliations:** 1 Department of Psychology, University of Warwick, Coventry, UK; 2 School of Social Work, Ariel University, Ariel, Israel

**Keywords:** mental health, rights of persons with disabilities, trauma, violence

## Abstract

**Aims:**

Previous research has shown that people with disabilities are disproportionately vulnerable to symptoms of psychological distress after exposure to armed conflict. Past work has also shown that individuals displaced by conflict are at heightened risk of post-traumatic stress. Using a national online sample of Ukrainians in the early weeks of the 2022 Russian invasion, we aim to examine associations between functional disability and symptoms of post-traumatic stress.

**Methods:**

We examined the association between levels of functional disability in the Ukrainian population and symptoms of post-traumatic stress during the 2022 Russian invasion of Ukraine. We analysed data from a national sample of 2000 participants from across this country, assessing disability using the 12-item World Health Organization Disability Assessment Schedule (WHODAS-12)(six domains of disability) and the International Trauma Questionnaire assessment of post-traumatic stress disorder (PTSD) symptomatology according to the Eleventh Edition of The International Classification of Diseases (ICD-11). Moderated regression examined the impact of displacement status on the disability–post-traumatic stress relationship.

**Results:**

Different domains of disability predicted post-traumatic stress symptoms (PTSSs) to varying extents, with overall disability score significantly associated with PTSSs. This relationship was not moderated by displacement status. Consistent with previous research, females reported higher levels of post-traumatic stress.

**Conclusions:**

In a study of a general population during a time of armed conflict, individuals with more severe disabilities were at greater risk of PTSSs. Psychiatrists and related professionals should consider pre-existing disability as a risk factor for conflict-related post-traumatic stress.

## Introduction

Conflict between Russia and Ukraine has been ongoing since 2014, with initial hostilities in Crimea and Donbas followed by a wider Russian invasion in February 2022. As has been seen in other military conflicts, this has had a serious deleterious impact on the mental health of affected populations (Charlson *et al*., 2019; Mesa-Vieira *et al*., 2022). Data collected in the early weeks of the 2022 invasion found that 52.7% of respondents reported psychological distress, while 54.1%, 46.8% and 12.1% indicated significant levels of anxiety, depression and insomnia, respectively (Xu *et al*., 2023). The ongoing conflict has also led to the substantial displacement of populations within Ukraine, with this population at risk of additional burden on their psychological health. A national survey of Ukrainian internally displaced people (2016) reported a post-traumatic stress disorder (PTSD) prevalence of 32% and 22% for depression and 22% and 17% for anxiety (Roberts *et al*., 2019). A study of elderly individuals conducted in 2016, during a period of active conflict in the aftermath of the annexation of Crimea (Summers *et al*., 2019), found that 34% of respondents living in Ukrainian government–controlled areas and 43% of respondents living in regions not under Ukrainian control showed symptoms of severe psychological distress. These figures should be viewed in the context of extremely high prevalence of mental illness rates in the country before both the current full-scale invasion and the earlier attacks on Crimea and the Donbas. Data collected in 2002 for the Ukraine World Mental Health Survey found lifetime mental illness prevalence of 31.6%, with Ukraine reporting one of the highest suicide rates in Europe (Bromet *et al*., 2005).

People with disabilities can face particularly significant negative mental health consequences after disaster and are at considerable risk following war-related trauma (Stough, 2010; Stough and Kelman, 2018). Data collected in 2002 from the Afghan population (Cardozo *et al*., 2004) found that 72% of respondents with disabilities reported depression, 85% anxiety and 42% PTSD. Those with chronic health conditions and disabilities were at the greatest risk of psychological distress. Consistent with studies elsewhere, which found that women exhibit higher levels of psychopathology during trauma (Brewin *et al*., 2000), women reported worse mental health than men. In a subsequent study of Afghan respondents (from 2004 to 2005) (Trani and Bakhshi, 2013), 86% of those with disabilities showed symptoms of potential mental distress using default K6 criteria, with again greater prevalence of distress amongst women.

In the context of the invasion of Ukraine, several researchers have made urgent calls for more data on the impact of the conflict on people with disabilities and for their situations to be given due attention (Patwary *et al*., 2022; Ruškus, 2022). However, despite the evidence of psychological trauma amongst the Ukrainian population and the high risk to mental health of people with disabilities exposed to armed conflict, we know of no previous work that has examined the association between disability severity and post-traumatic stress symptoms (PTSSs) in a general population affected by armed conflict. To this end, we assessed associations between disability and PTSSs in the population employing a web-based sample which aimed to be representative in terms of age and gender, as well as geographical region prior to any displacement. We hypothesise that (a) severity of disability will predict severity of PTSSs and (b) females will experience higher rates of PTSSs (Brewin *et al*., 2000). Previous studies indicate that internally displaced people and refugees are systematically more vulnerable to various forms of psychological distress than people who have not been displaced in conflict (Blackmore *et al*., 2020), and we expect to replicate this trend. We also explore the extent to which this displacement will moderate any association between disability and post-traumatic stress.

## Methods

### Recruitment and sample

We collected data in the first weeks after the invasion, during which attacks from Russia occurred across the whole country. We report analysis of secondary data from an online sample of 2000 people aged 18–55 surveyed in Ukraine between 7 and 15 April 2022 (*M* age 37.18 years, SD = 9.3), 1026 (51.3%) female (see also Ben-Ezra *et al*., 2023). We used an existing panel maintained by the Ukrainian branch of the survey company Kantar, aiming at obtaining representation of age, sex and region (before displacement). Data were collected in accordance with the Strengthening the Reporting of Observational Studies in Epidemiology (STROBE) guidelines for observational studies (von Elm *et al*., 2008).

Each participant received a digital invitation and provided electronic informed consent. Inclusion criteria were age (18–55) and fluency in Ukrainian. Using G*Power (v. 3.1.9.4) (Faul *et al*., 2007, 2009), we estimated a sample of 1975 participants to be required to detect low-medium effect sizes of 0.20, with 99% power and a 1% significance level. Of 2765 who clicked through to the survey, 176 (6.4%) were omitted for failing to meet inclusion criteria, a further 326 (11.8%) dropped out and 263 (9.5%) were removed to meet quotas for representative sampling. Of our 2000 respondents, 1455 individuals (72.8%) were not displaced, 389 (19.4%) displaced inside of Ukraine and 156 (6.6%) displaced outside of Ukraine. In terms of the major Ukrainian regions of origin, 569 individuals (28.5%) were from the East, 358 (17.9%) from the West, 483 (24.2%) from Kyiv, 160 (8%) from the North, 217 (10.8%) from the Centre and 213 (10.6%) from the South. Further sample descriptives are presented in Table 1. The dataset is available on the Open Science Framework (https://osf.io/z5adg/).

**Table 1.** Descriptive statistics

Variable	Means (*M*, SD)/Frequencies (*N*, %)
Age	*M* = 37.18 (9.23)
Sex (female)	*N* = 1026 (51.3)
Displaced	*N* = 1455 (72.8)
Not displaced	*N* = 545 (27.2)
ITQ score (Post-traumatic stress symptoms)	*M =* 11.64 (4.91)
WHODAS domain (range 1−5): getting around	*M =* 1.71 (1.78)
WHODAS domain: self-care	*M* = 1.07 (1.67)
WHODAS domain: getting along with people	*M =* 1.71 (1.81)
WHODAS domain: life activities	*M* = 2.00 (1.82)
WHODAS domain: participation in society	*M* = 2.17 (1.87)
WHODAS domain: understanding and communicating	*M* = 1.83 (1.77)
WHODAS total	*M* = 10.51 (8.65)

### Research ethics approval statement

All participants provided informed consent. Ethical approval for original data collection was provided by the University Ethics Committee of the final author (AU-SOC-YHR-20220311). Approval for this re-analysis was provided by the Ethics Committee of the first author (University of Warwick, Psychology: PGR_21-22/35). All aspects of this research were performed in accordance with the principles of the Declaration of Helsinki (World Medical Association, 2013).

### Measures

*PTSSs* were assessed using six items from the International Trauma Questionnaire (ITQ) (Cloitre *et al*., 2018) previously validated for use with the Ukrainian population (Shevlin *et al*., 2018). Participants were asked how much they were bothered by each of six symptoms (e.g. ‘feeling jumpy or easily startled’) in the previous month and responded on a five-point Likert scale ranging from not at all (0) to extremely (4) (*α* = 0.86). Possible scores ranged between 0 and 24.

*Disability status* was measured using the 12-item World Health Organization Disability Assessment Schedule (WHODAS) 2.0 (Üstün *et al*., 2010). The WHODAS 2.0 conceptualizes disability in accordance with the International Classification of Functioning (ICF) (World Health Organization, 2001) and encourages a holistic view of individual functioning across different domains. In line with current sociological thinking in disability studies (Shakespeare, 2017), we chose to focus on individual functioning, regardless of diagnostic labels. This tool has previously demonstrated reliability and validity in international use (Federici *et al*., 2017), although only the full 36-item version has been previously used in the Ukrainian context. The 12 items measure difficulties in six different domains of functioning, with two items per domain: (1) Cognition; (2) Mobility; (3) Self-care; (4) Getting along with others; (5) Life activities and (6) Participation (overall *α* = 0.91). Respondents indicated the level of difficulty they had with each item using a five-point Likert scale, from none (1) to extreme or cannot do (5). We used the simple scoring method (Üstün *et al*., 2010) adding together scores to create a single overall score (from 12 [no disability] to 60 [complete disability]).

To assess *displacement*, respondents were coded as *Displaced* if they either had been forced to move within Ukraine as result of the conflict or had to leave the country because of the war.

### Analyses

To assess associations between disability and PTSS, we initially ran Pearson correlations between each subscale of the WHODAS-12 and the total post-traumatic stress score from the ITQ. We then ran a moderated regression with WHODAS-12 total score as the focal predictor and post-traumatic stress score as the outcome. Displacement status was used as the moderator, with sex as a covariate. All analyses were conducted using SPSS version 27. Moderated regression was conducted using the process macro (Hayes, 2022) with a heteroscedasticity-consistent standard error estimator (Hayes and Cai, 2007).

## Results

Mean PTSS score was 11.64 (range 0–24, SD = 4.91) and PTSD prevalence was 30.8%. Mean disability (WHODAS-12) score was 10.51 (possible range = 12–60, SD = 8.65). Average WHODAS-12 (disability) subscale scores ranged from 1.07 (SD = 1.67) for self-care to 2.17 (SD = 1.87) for participation in society. There were moderate positive zero-order correlations between each of the disability measures and PTSSs. All six of the disability sub-scales were significantly associated with PTSD symptoms as demonstrated by the Spearman correlation coefficients of 0.30 (self-care)–0.45 (participation in society), all *P* < 0.001) (Table 2).

**Table 2.** Correlations between WHODAS sub-scales and ITQ total score

		WHODAS getting around	WHODAS self-care	WHODAS getting along with others	WHODAS life activities	WHODAS participation in society	WHODAS understanding and communicating
WHODAS self-care	*r*	0.56^**^					
	Sig.	0.000					
	*N*	2000					
WHODAS getting along with others	*r*	0.45^**^	0.56^**^				
	Sig.	0.000	0.000				
	*N*	2000	2000				
WHODAS life activities	*r*	0.60^**^	0.58^**^	0.59^**^			
	Sig.	0.000	0.000	0.000			
	*N*	2000	2000	2000			
WHODAS participation in society	*r*	0.58^**^	0.52^**^	0.57^**^	0.62^**^		
	Sig.	0.000	0.000	0.000	0.000		
	*N*	2000	2000	2000	2000		
WHODAS understanding and communicating	*r*	0.59^**^	0.56^**^	0.56^**^	0.68^**^	0.68^**^	
	Sig.	0.000	0.000	0.000	0.000	0.000	
	*N*	2000	2000	2000	2000	2000	
ITQ total	*r*	0.34^**^	0.30^**^	0.34^**^	0.40^**^	0.45^**^	0.43^**^
	Sig.	0.000	0.000	0.000	0.000	0.000	0.000
	*N*	2000	2000	2000	2000	2000	2000

** *P* < 0.01 level (two-tailed).

In line with our first hypothesis, a moderated regression model (Table 3) indicated that increasing levels of functional disability in the Ukrainian population predicted greater endorsement of PTSSs (*β* = 0.27, *t* = 21.67, *P* = < 0.001). Also, as anticipated, displacement was significantly associated with PTSSs, although the disability–PTSSs relationship was not moderated by displacement (*β* = −0.01, *t* = −0.43, *P* = 0.67). Women exhibited more PTSSs (*β* = 1.71, *t* = 8.88, *P* = < 0.001).

**Table 3.** Moderated linear regression on disability and post-traumatic stress symptoms

Predictor	*Β*	Standard error	*t*	*P*	95% confidence interval
Constant	8.83	0.54	16.40	<0.001	7.78, 9.89
Sex	1.71	0.19	8.88	<0.001	1.33, 2.08
Disability	0.27	0.01	21.67	<0.001	0.22, 0.27
Displacement	0.43	0.15	2.83	0.0046	0.13, 0.73
Interaction (disability × displacement)	−0.01	0.02	−0.43	0.66	−0.05, 0.03

*β* = standardized beta coefficient

## Discussion

In this paper, we present one of the first analyses to examine associations between disability severity and PTSSs during a period of war. We find that women and people with more severe disabilities endorsed greater PTSSs, as do those who are displaced, but there was no moderation of the disability–post-traumatic stress relationship by displacement.

A variety of disabilities including vision impairment (Bonsaksen *et al*., 2022), physical disability (Turner *et al*., 2006) and autism spectrum disorders (Lai *et al*., 2019) have each been previously associated with increased vulnerability to PTSD. The associations between PTSSs and all six dimensions of the WHODAS-12 suggest that increasing disability severity may make individuals more vulnerable to post-traumatic stress during a period of conflict. At the same time, there were differences in associations between disability and PTSSs by disability domain. Self-care was the dimension most weakly associated with post-traumatic stress, while impairments related to social functioning had the strongest association. This may be because respondents with those impairments which make self-care more difficult may not be available when sampling a general population, given that the Ukrainian health and social care system is most likely to place these people in an institutional setting.

Previous research (Blackmore *et al*., 2020; Johnson *et al*., 2021) has suggested that refugees and internally displaced people are at heightened risk for a variety of mental health problems when compared to individuals who have not been displaced. While we did find that displacement status was associated with PTSSs, this did not have a moderating role in the disability–post-traumatic stress relationship. This may be because of a lack of possibility for movement amongst refugees with disability. While housing, employment and access to health and support services were already problematic for Ukrainians with disabilities living in the Donetsk and Luhansk Oblasts in the east of the country (Gazizullin and Solodova, 2019), it may have been particularly difficult for people with disabilities in these populations to move. Indeed, previous research on predictors of mobility among Ukrainians after the annexation of Crimea in 2014 (Mykhnenko *et al*., 2022) finds that people with disabilities were less likely to leave their homes than their nondisabled counterparts. Mobility may also be challenging for those suffering with cognitive challenges (e.g., inability to concentrate) or high levels of social anxiety who may feel less able and confident to leave familiar surroundings in favour of an unknown, albeit potentially safer, destination. Our finding that female gender is associated with greater endorsement of PTSSs is consistent with previous studies of populations exposed to war-related trauma (Charlson *et al*., 2016).

Our study has several strengths. We present a novel examination of disability and PTSD symptoms during conflict employing the widely used WHODAS 2.0 12-item measure of disability. We note that our disability scores measured using WHODAS 2.0 in the Ukrainian population five weeks after the Russian invasion were only slightly higher than previously established norms for the Polish population using the same scoring method, during peacetime (Cwirlej-Sozanska *et al*., 2020). This suggests that our measure accurately depicts the prevalence of pre-existing functional impairment in the Ukrainian population and is not overly biased by the presence of newly acquired disabilities caused by the conflict.

At the same time, we recognize several limitations. First, this study was of the Ukrainian general population. There are substantial differences in the treatment of people with and without disabilities in Ukraine. As a result of the Soviet past – and continued influence of Soviet and Russian traditions even following the independence of Ukraine – people with disabilities do not have equal rights (Phillips, 2009) with many placed in long-term institutional care (Shaw, 2022). This means that people with especially severe disabilities who we might infer would be at the highest levels of risk from PTSD after being exposed to the war were largely inaccessible for the study. Second, because we assessed PTSD symptoms in the early weeks of the Russian invasion, we focused on PTSSs using the International Trauma Questionnaire but did not assess PTSD symptoms prior to the invasion. Third, because we take the approach that diagnostic labels are less important than levels of functioning across the different domains under consideration, we did not explicitly ask about types of disabilities. This meant we were unable to identify any specific disabilities associated with PTSD symptomology. We recognize that future work might also fruitfully complement our current approach with more detail about specific disabilities. Finally, we excluded participants over the age of 55 due to their more limited use of the internet in Ukraine. This nevertheless restricted our analyses of age effects on PTSD symptoms.

Our study has several implications for practitioners. First, we add to the existing literature on war trauma and disability by showing that a range of disability/functional impairments may place patients at risk of post-traumatic stress. Given this range of risk factors, health professionals should take account of the broader infrastructure and environmental damage caused by conflict and its implications for social functioning as well as daily care for many people with disabilities (Racioppi *et al*., 2022). Practitioners need to make use of evidence-based strategies for improving psychosocial health in emergencies (Weissbecker *et al*., 2023) with particular reference to the unique needs of people with different disabilities. In this case, medical personnel may also wish to direct people with disabilities to services and support groups that not only assist in meeting their basic needs but provide opportunities for building or augmenting a social support network. This support is likely to be important for people with disabilities in general (Honey *et al*., 2011) and those relocated as a result of conflict.

## Data Availability

The dataset is available on the Open Science Framework (https://osf.io/z5adg/).
